# Supplementary figures and images for: G protein-coupled receptor SmGPCR9 interacts with neuropeptides and controls spermatogenesis in Schistosoma mansoni
Source: PLoS Pathog. 2026 Jul 7;22(7):e1014096. doi: 10.1371/journal.ppat.1014096 (PMC13367911; doi:10.1371/journal.ppat.1014096)

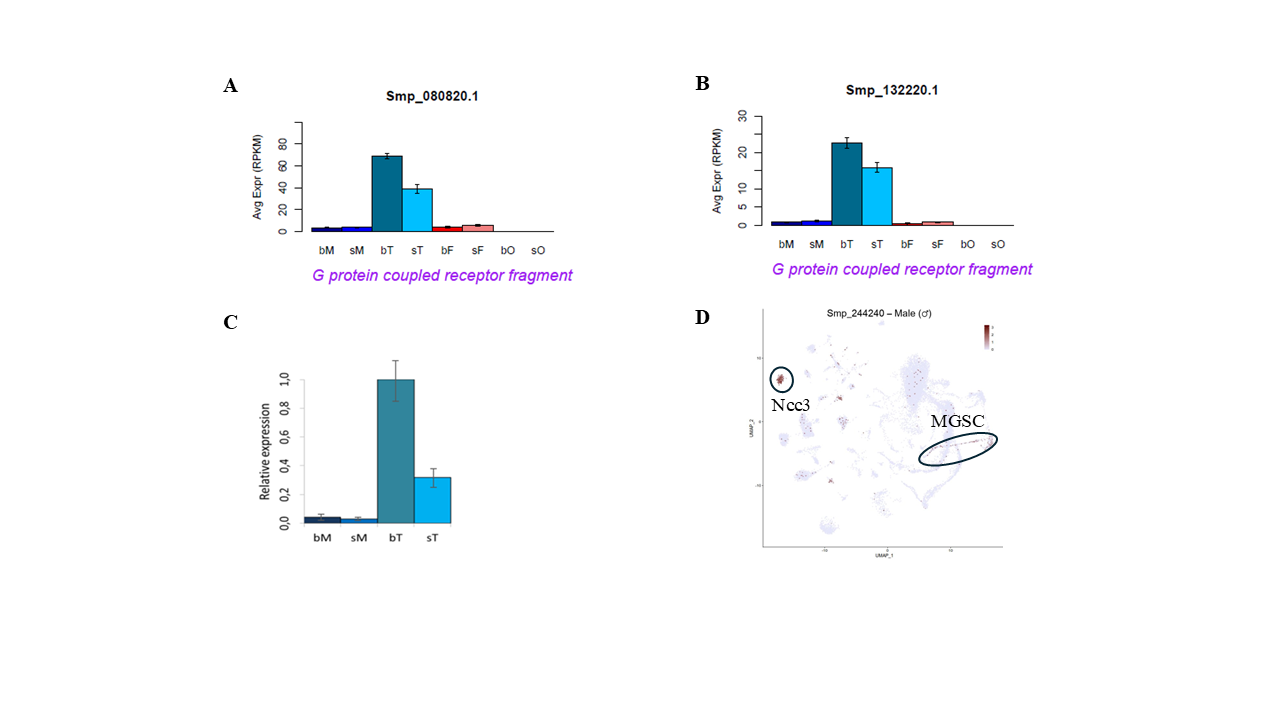

Supplement: S1 Fig — A-B, Former data from bulk RNAseq analyses of adult S. mansoni and their gonads identified two Smp numbers, Smp_080820.1 and Smp_132220.1, for Smgpcr9, based on version 5 of the genome (42). Recent genome updates, versions 7 and 10, provided the new number Smp_244240 for this gene (55; https://parasite.wormbase.org/Schistosoma_mansoni_prjea36577/Info/Index/); C, RT-PCR analysis confirmed the testes-preferential and pairing-influenced transcript profile of Smgpcr9 in males. D, Single-cell RNAseq showed preferential expression of Smp_244240 in late male germ cells (MGSC) and the neuronal cell cluster 3 (Ncc3) of S. mansoni males (56). Abbreviations: bM, males with pairing experience; sM, males without pairing experience; bT, testes of bM; sT, testes of sM; bF, females with pairing experience; sF, females without pairing experience; bO, ovaries of bF; sO, ovaries of sF; Ncc, neuronal cell cluster. (TIF) [file ppat.1014096.s001.tif]

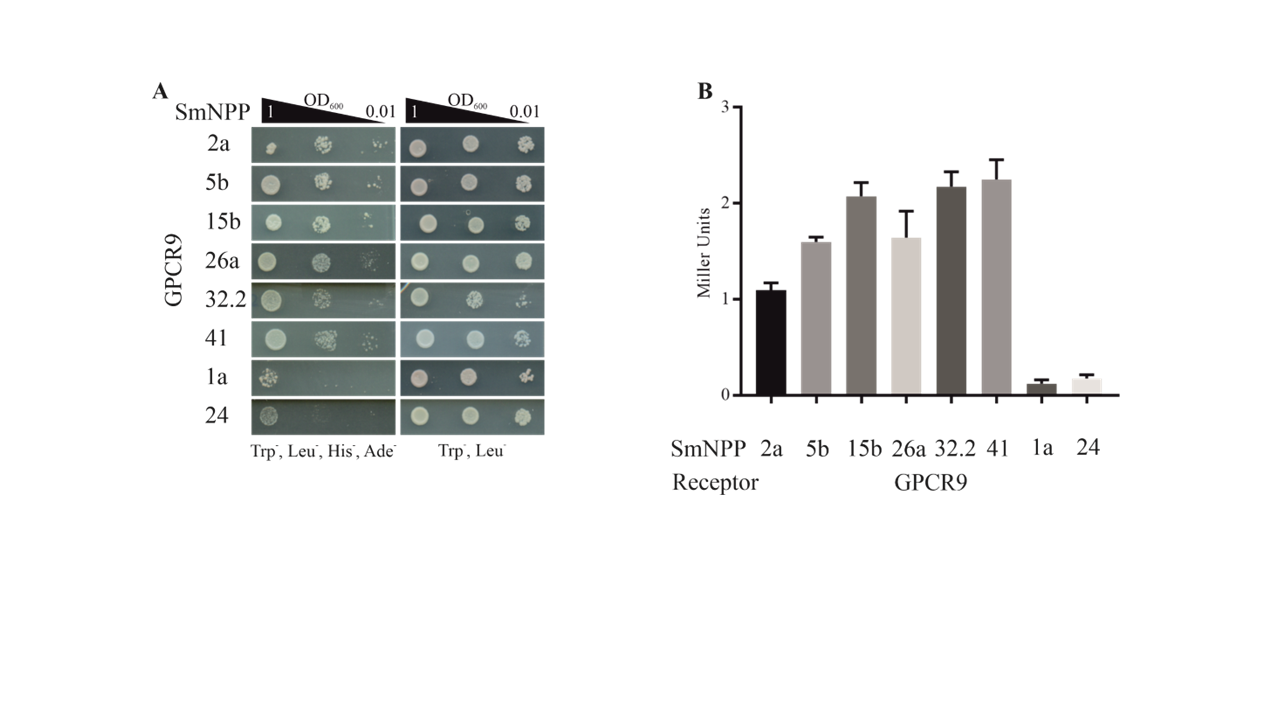

Supplement: S2 Fig — A, MALAR-Y2H assay to detect protein-protein interactions between Smgpcr9 and neuropeptides (NPPs) of S. mansoni. Shown are results of cell growth assays of yeast strain AH109 transfected with plasmids expressing ligand fusion proteins (NPPs), which was mated with yeast strain Y187 transfected with plasmids expressing Smgpcr9 (57). Three different OD600 concentrations of diploid yeast cells were dropped onto SD/Trp− Leu − His − Ade − and Trp − Leu − plates, which served as growth control. Colony growth was monitored after 48 and 72 h, respectively. B, ONPG-assays to determine β-Gal activity of diploid cells (as in A; 57) showed strongest interactions for SmNPPs 2a, 5b, 15b, 26a, 32.2, and 41, whereas 1a and 24 appeared to be weak putative interaction partners. Shown are the mean values of two clones (n = 2; 57). (TIF) [file ppat.1014096.s002.tif]

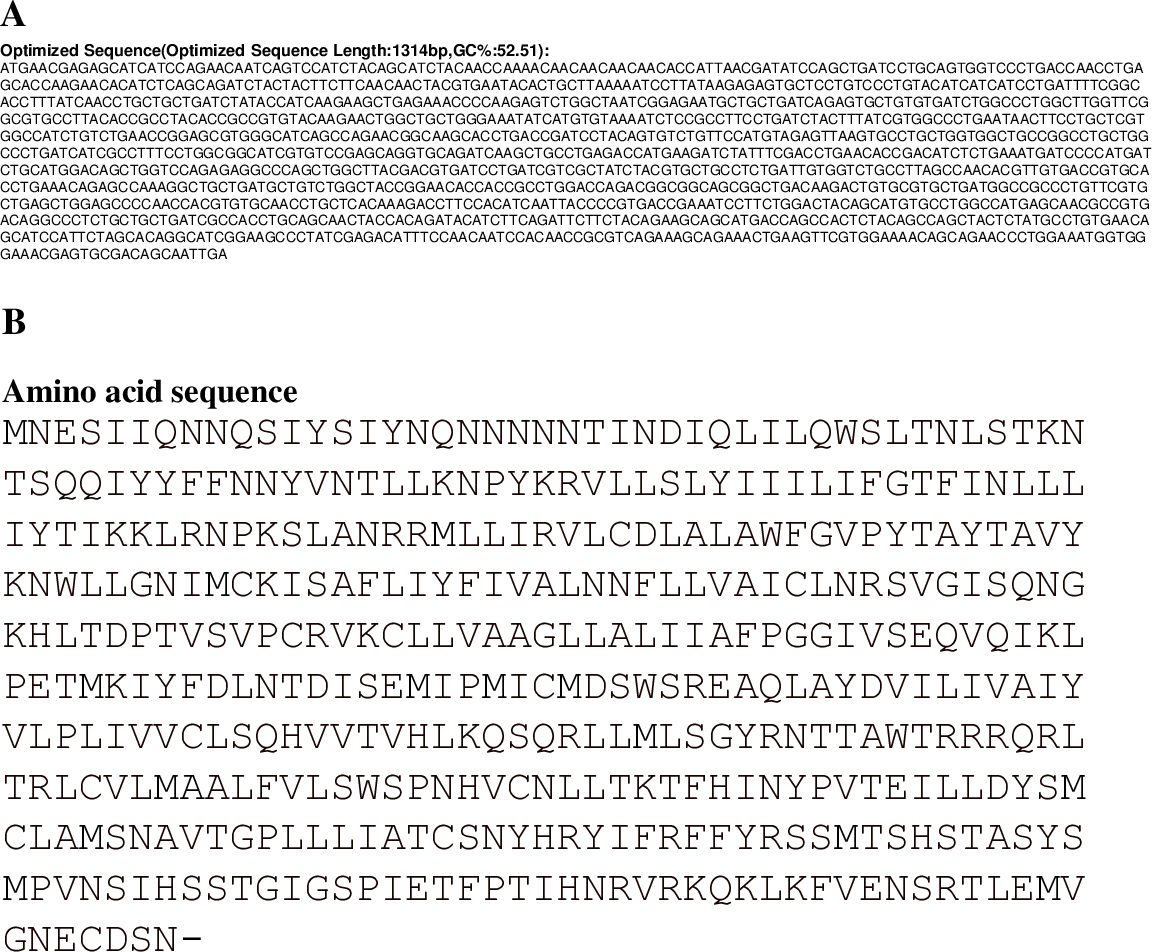

Supplement: S3 Fig — The Smgpcr9 coding sequence (CDS) of S. mansoni was adapted to human codon usage to optimise expression in HEK293-6E cells. Shown here are the optimised Smgpcr9 CDS (A) and the corresponding amino acid sequence (B). (TIF) [file ppat.1014096.s003.tif]

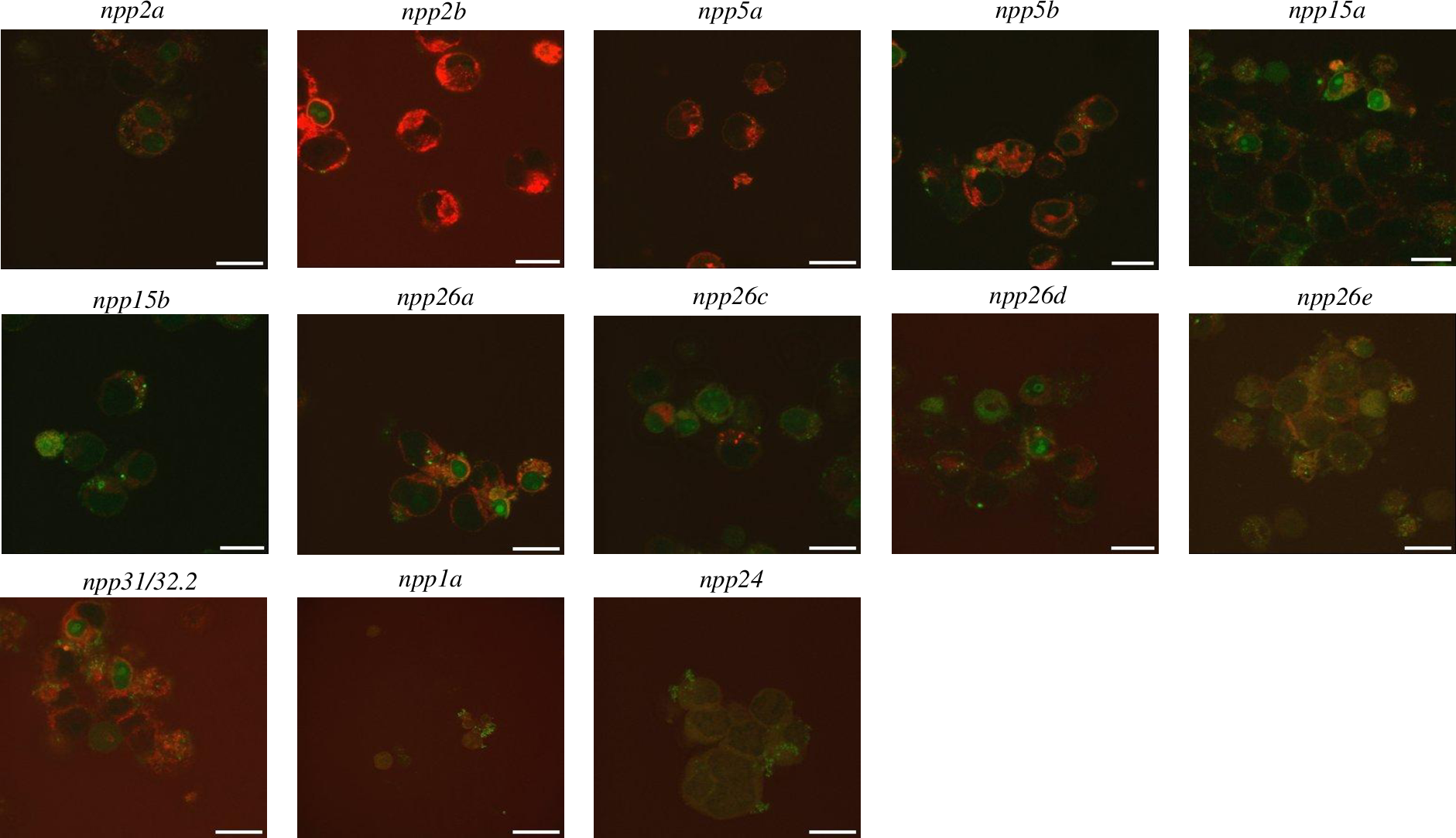

Supplement: S4 Fig — HEK293-6E cells transiently expressing pCDNA_mCitrine_GPCR9_dsRed were stimulated with the selected, soluble NPPs for 30 min at 37⁰C. Colocalizing signals were observed as internalized dots. SmNPP11a and SmNPP24 failed to show internalization signals. Scale bars = 30 µm. Experiments were performed in two independent biological replicates. (TIF) [file ppat.1014096.s004.tif]

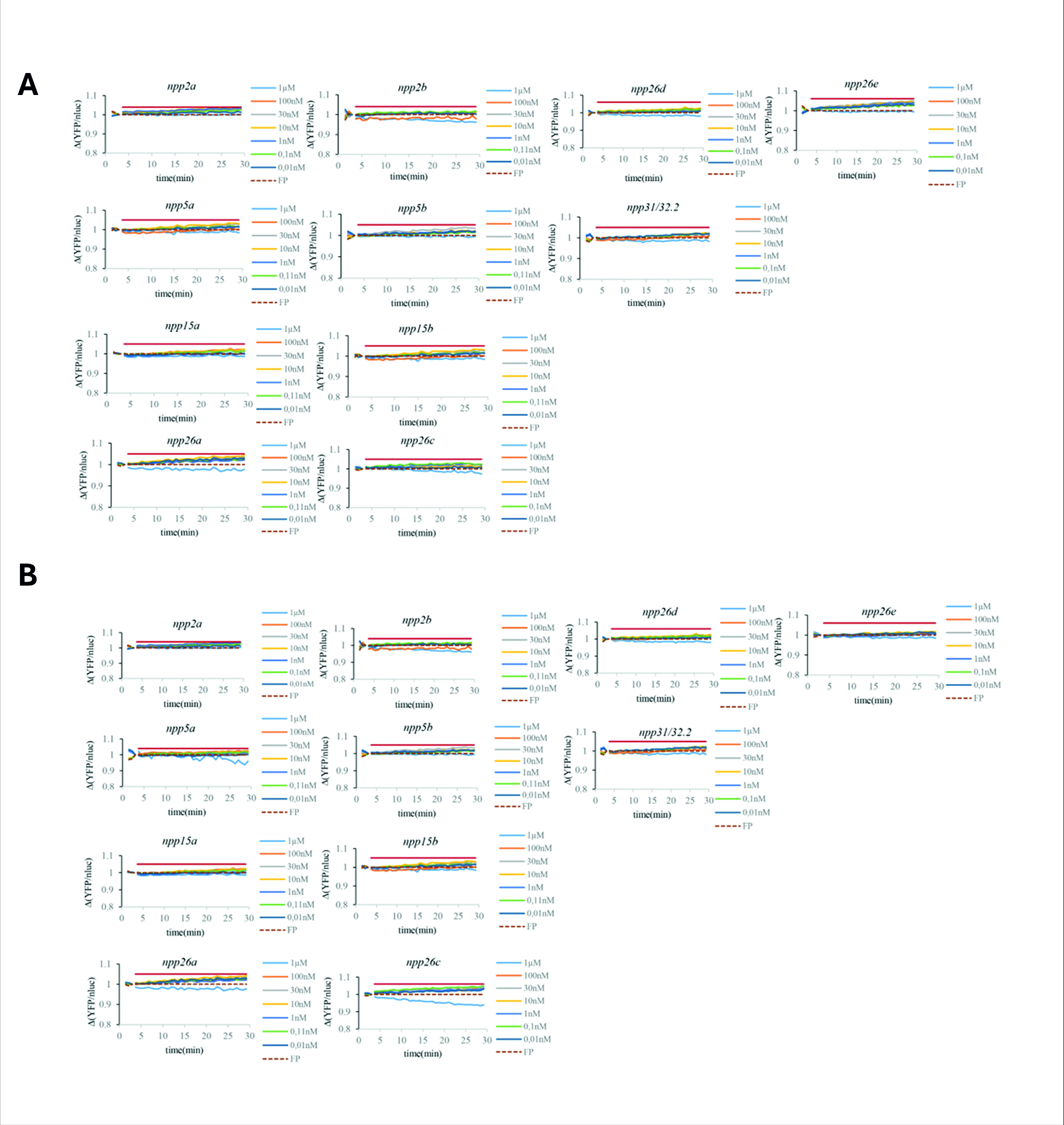

Supplement: S5 Fig — Schematic representations of the same Gαq activation assays showing specific protein–protein interactions of SmGPCR9 with SmNPPs (as indicated). In A, the tagged version of SmGPCR9 was used and in B the untagged version. SmNPPs 26b and 41, which showed highest evidence for interaction, are part of Fig 1. (TIF) [file ppat.1014096.s005.tif]

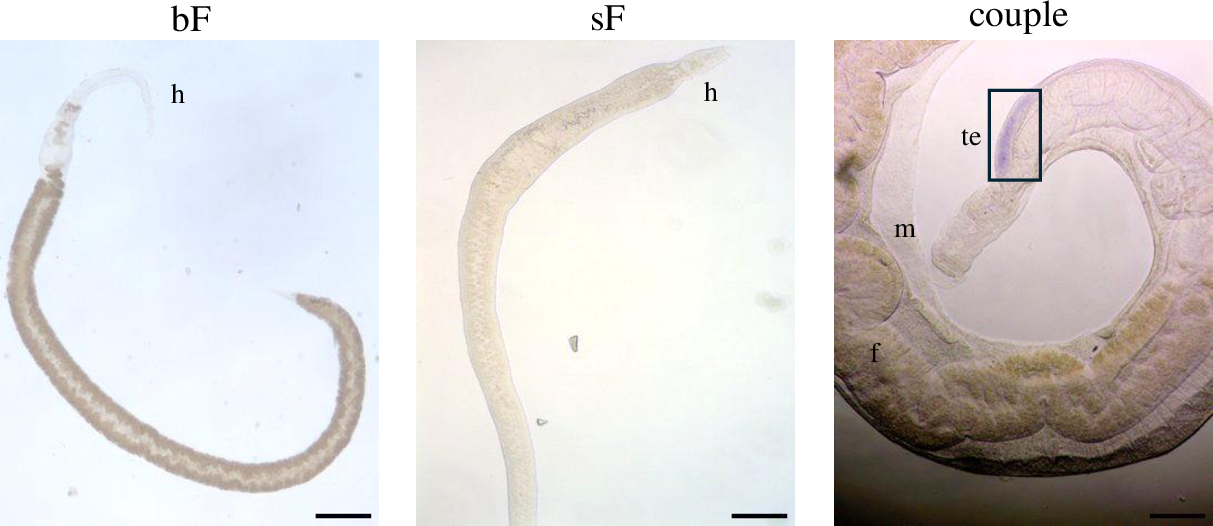

Supplement: S6 Fig — No clear signals were observed upon WISH in bisex females (bF), single-sex females (sF), and in paired females (see couple), whereas Smgpcr9 transcripts were observed in male testes, as expected (see couple). Scale bars = 200 µm. Abbreviations: te, testes; h, head (anterior) part; m, male; f, female. (TIF) [file ppat.1014096.s006.tif]

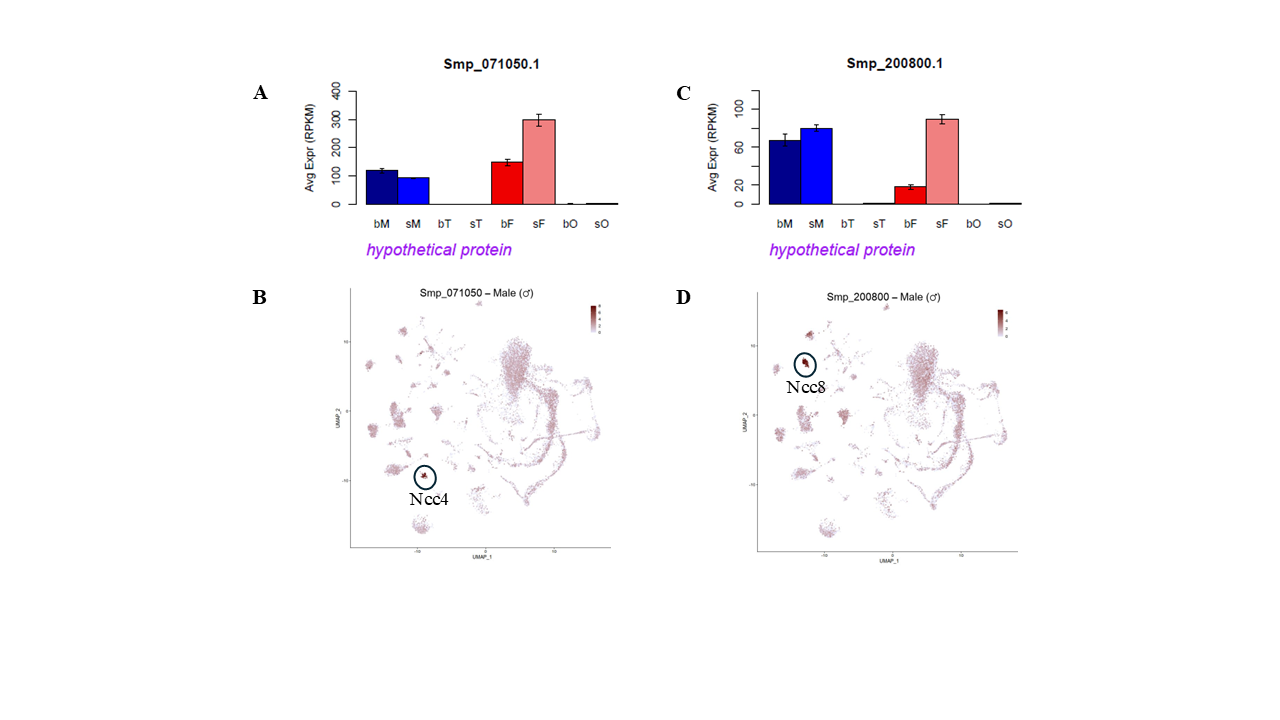

Supplement: S7 Fig — According to the previous bulk RNA-seq study of adult S. mansoni and their gonads, Smnpp26 (Smp_071050; A) and Smnpp41 (Smp_200800; C) are mainly transcribed in adult worms but not their gonads, with a pairing-dependent expression profile in females, and a bias for unpaired females. Single cell RNA-seq data exhibited dominant expression for Smnpp26 in neuronal cell cluster 4 (Ncc4; B), whereas Smnpp41 dominates in neuronal cell cluster 8 (Ncc8; D) (56). Abbreviations: bM, males with pairing experience; sM, males without pairing experience; bT, testes of bM; Ncc, neuronal cell cluster; sT, testes of sM; bF, females with pairing experience; sF, females without pairing experience; bO, ovaries of bF; sO, ovaries of sF. (TIF) [file ppat.1014096.s007.tif]

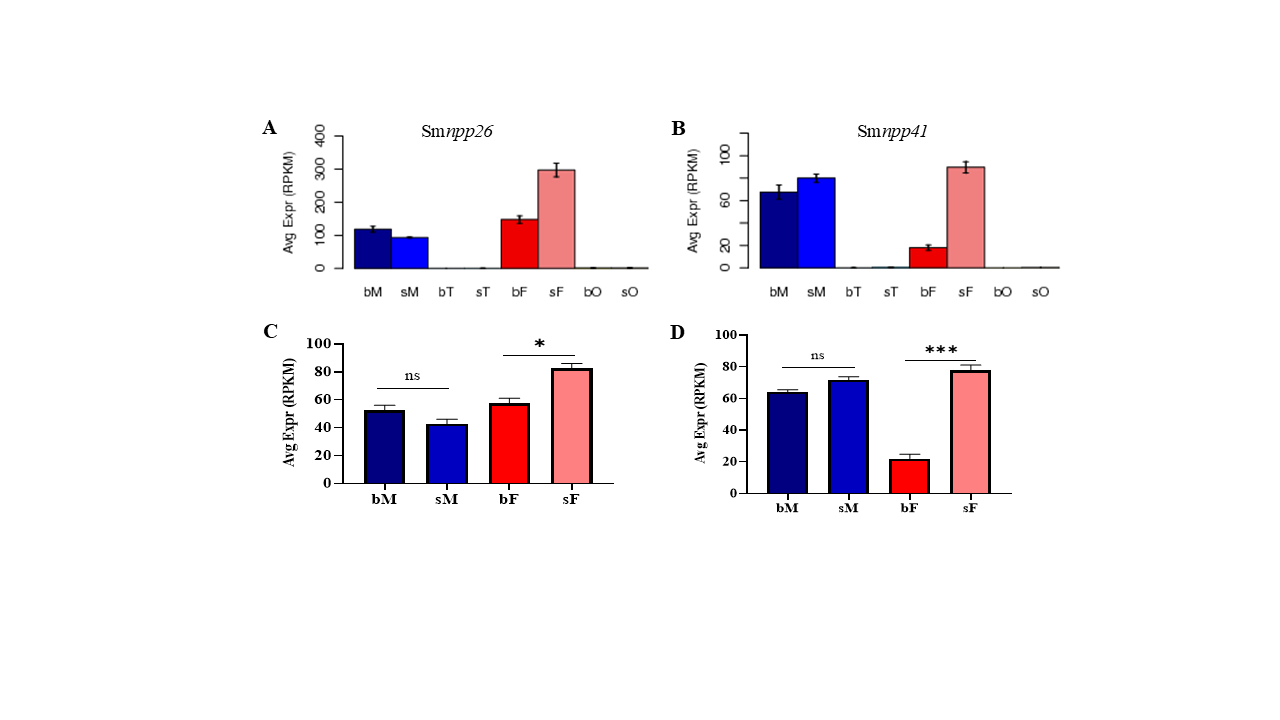

Supplement: S8 Fig — Shown are transcript profiles of Smnpp26 (A), and Smnpp41 (B) obtained by bulk RNA-seq analysis of female and male S. mansoni and their gonads (42). RT-qPCR confirmed the transcript patterns of Smnpp26 (C), and Smnpp41 (D) including their pairing-influenced transcriptions in females. Abbreviations: bM, bisex males (pairing-experienced); sM, single-sex males (pairing-unexperienced); bT, testes of bM; sT, testes of sM; bF, bisex females (pairing-experienced); sF, single-sex females (pairing-unexperienced); bO, ovaries from bF; sO, ovaries from sF. Average expression (Avg Expr) was based on RPKM (Reads Per Kilobase per Million mapped reads) values. Significant differences were determined by t-test and indicated as: ***P < 0.001, **P < 0.01, *P < 0.05, ns, no significance. (TIF) [file ppat.1014096.s008.tif]

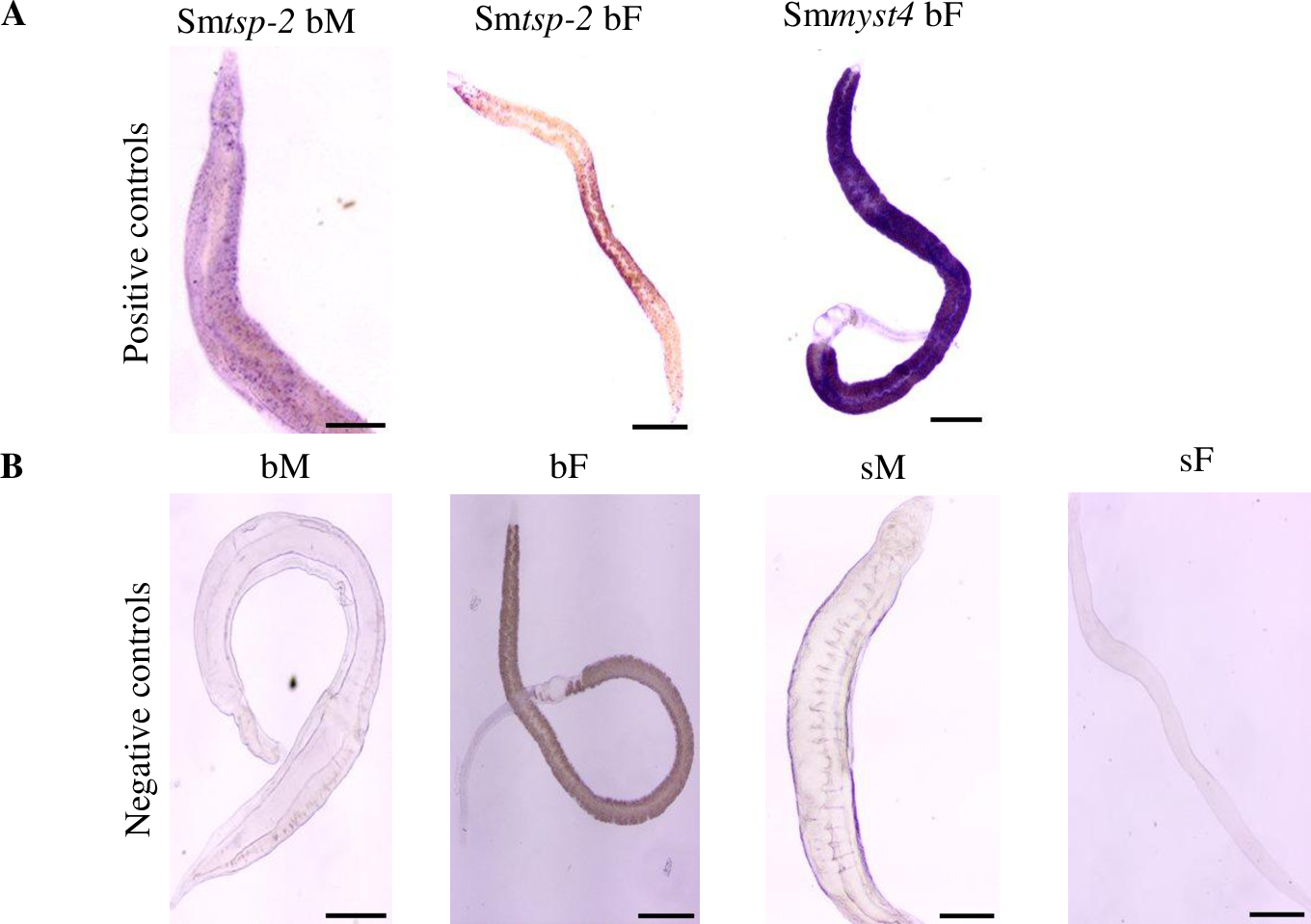

Supplement: S9 Fig — A, As positive controls for WISH, we used the tegumentally expressed Smtsp-2 (62) and the vitellarium-specifically expressed Smmyst4 (32); all of which showed expected transcript patterns. B, As negative controls, we used sense probes of the genes, which showed no signals upon hybridisation. Scale bars = 200 µm. (TIF) [file ppat.1014096.s009.tif]

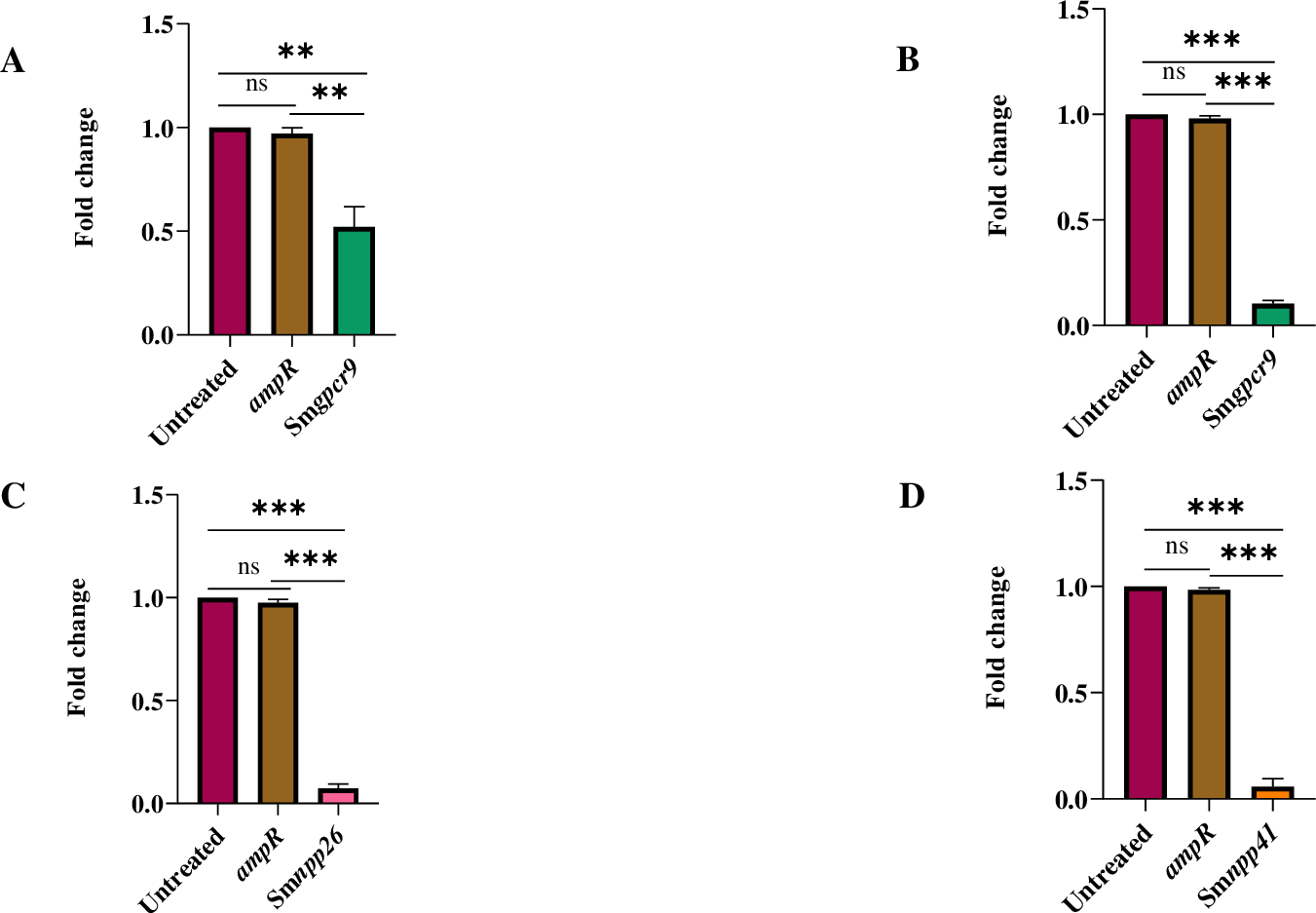

Supplement: S10 Fig — For this experimental approach, male cDNA was used as template for RT-qPCR. A, Compared to the controls (untreated, no dsRNA; ampR, irrelevant dsRNA), using a single dsRNA resulted in the reduction of the Smgpcr9 transcript level of about 50% after 15 days in vitro treatment. B, Using the two-probes/per-target approach, KD efficiency of Smgpcr9 was 90% ± 3%. For Smnpp26 RNAi (C) and Smnpp41 RNAi (D), using single dsRNAs in each case resulted in KD efficiencies of 94% ± 2%, and 93% ± 2%, respectively. Significant differences were determined by t-test and indicated as: ***P < 0.001, **P < 0.01, *P < 0.05. (TIF) [file ppat.1014096.s010.tif]

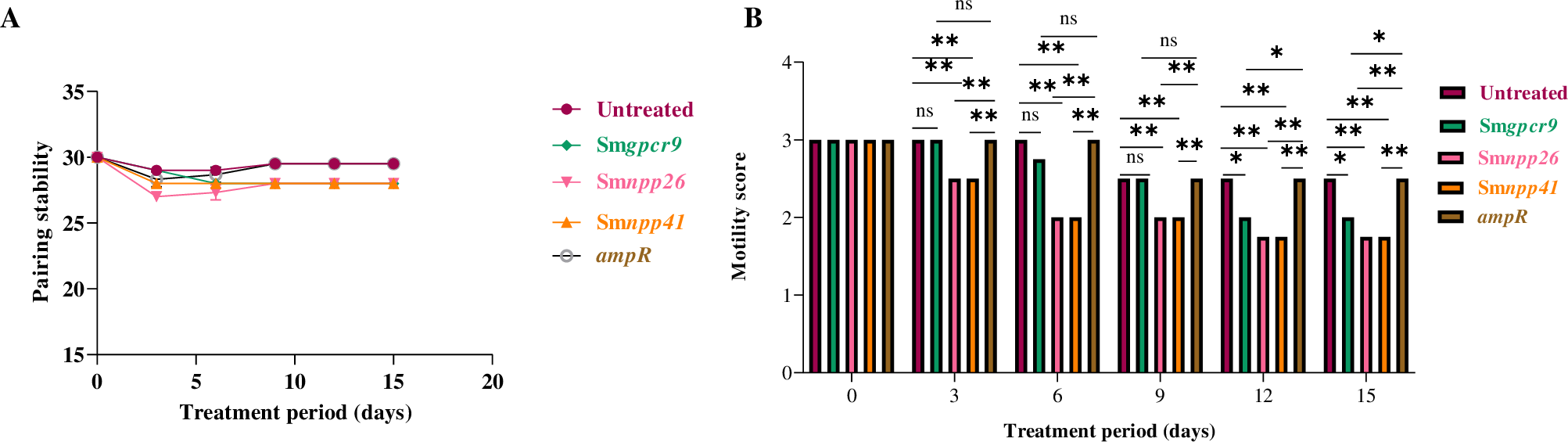

Supplement: S11 Fig — Untreated S. mansoni couples (without dsRNA) and couples treated with irrelevant ampR dsRNA served as controls. All worms were kept under the same in vitro-culture conditions for 15 d. A, We observed no effect for pairing stability. B, Compared to the controls, motility was significantly reduced for all target genes (as indicated) but at different time points, with Smgpcr9 showing the slowest significant effect on motility following RNAi. There were no distinct effects observed with ampR dsRNA treatment as shown in figures. Significant differences were determined by t-test and indicated as: ***P < 0.001, **P < 0.01, *P < 0.05. (TIF) [file ppat.1014096.s011.tif]

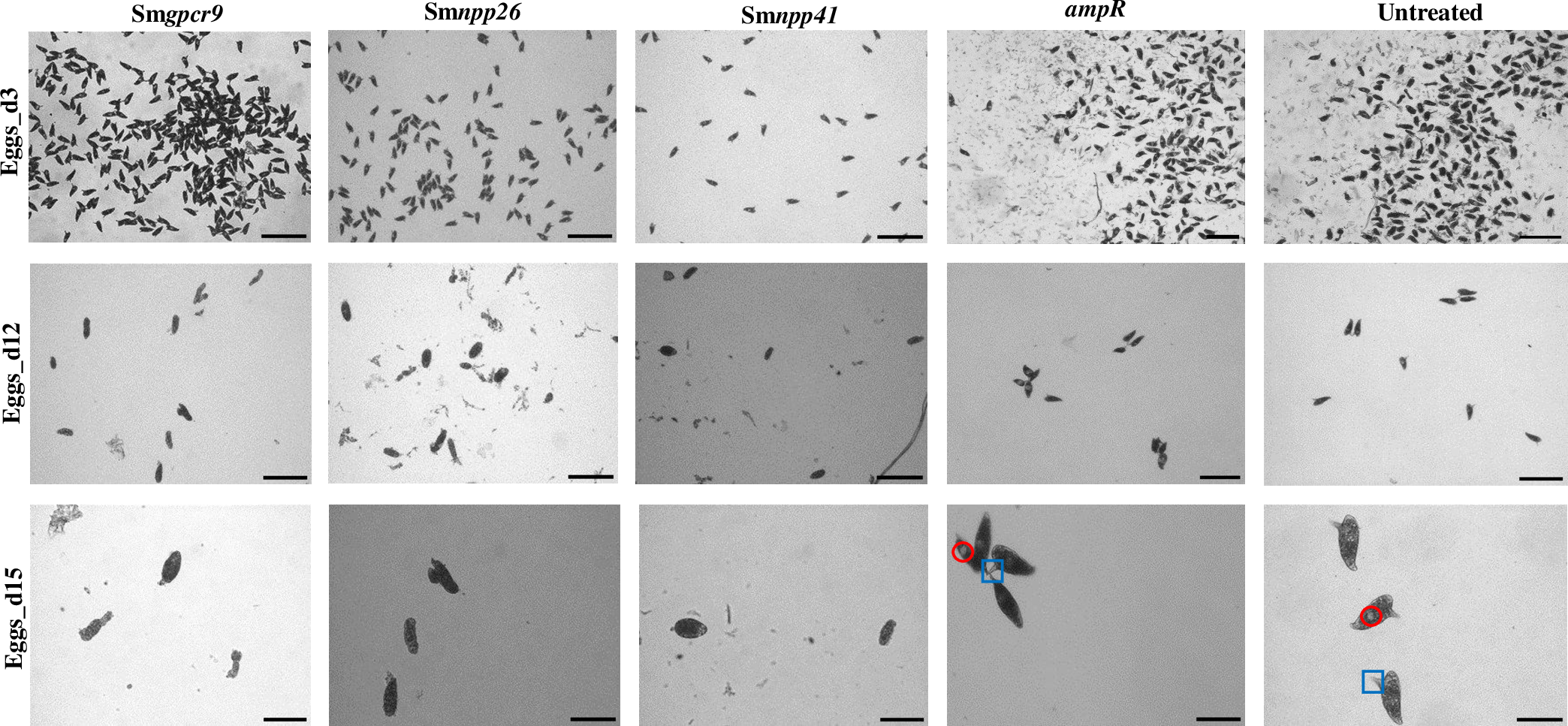

Supplement: S12 Fig — Starting between days 9–12 after dsRNA treatment, deformed eggs were produced in all RNAi groups, as indicated, except the control groups (ampR and untreated). Even after 15 days, worms of both control groups produced eggs of normal size with visible zygotes (red circles) and normal spines (blue squares). In contrast, eggs of worms of all RNAi groups showed various defects like size reduction, missing spines, and/or no zygotes. (TIF) [file ppat.1014096.s012.tif]

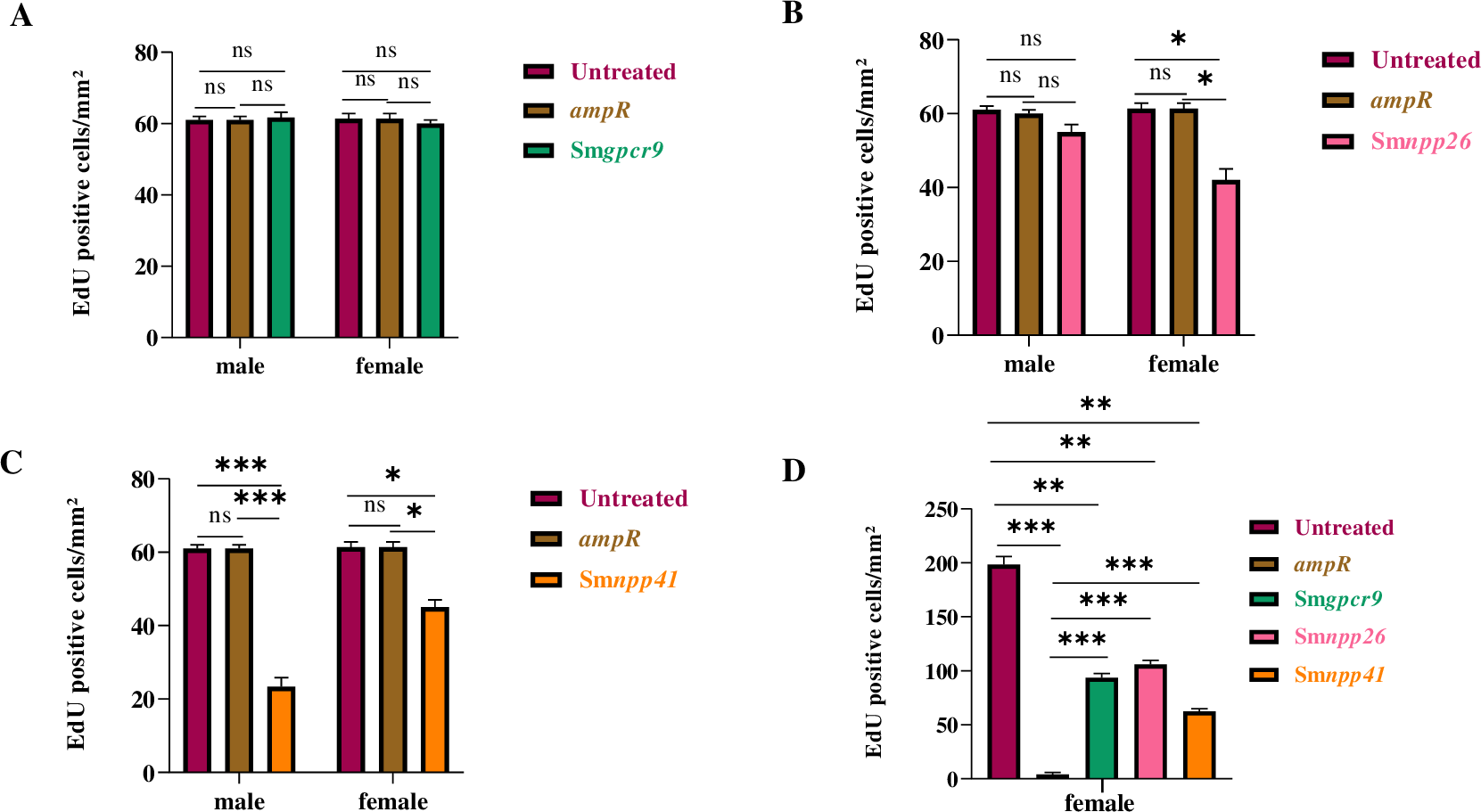

Supplement: S13 Fig — Image J-based quantification of the amounts of EdU-stained cells in the gonads of males (testes) and females (ovary) treated with dsRNA against (A) Smgpcr9 (B) Smnpp26, and (C) Smnpp41 versus controls (untreated and ampR dsRNA- treated). EdU quantification was also done for the vitellarium of females, showing minimal fluorescence only for females following ampR dsRNA treatment (D). (TIF) [file ppat.1014096.s013.tif]

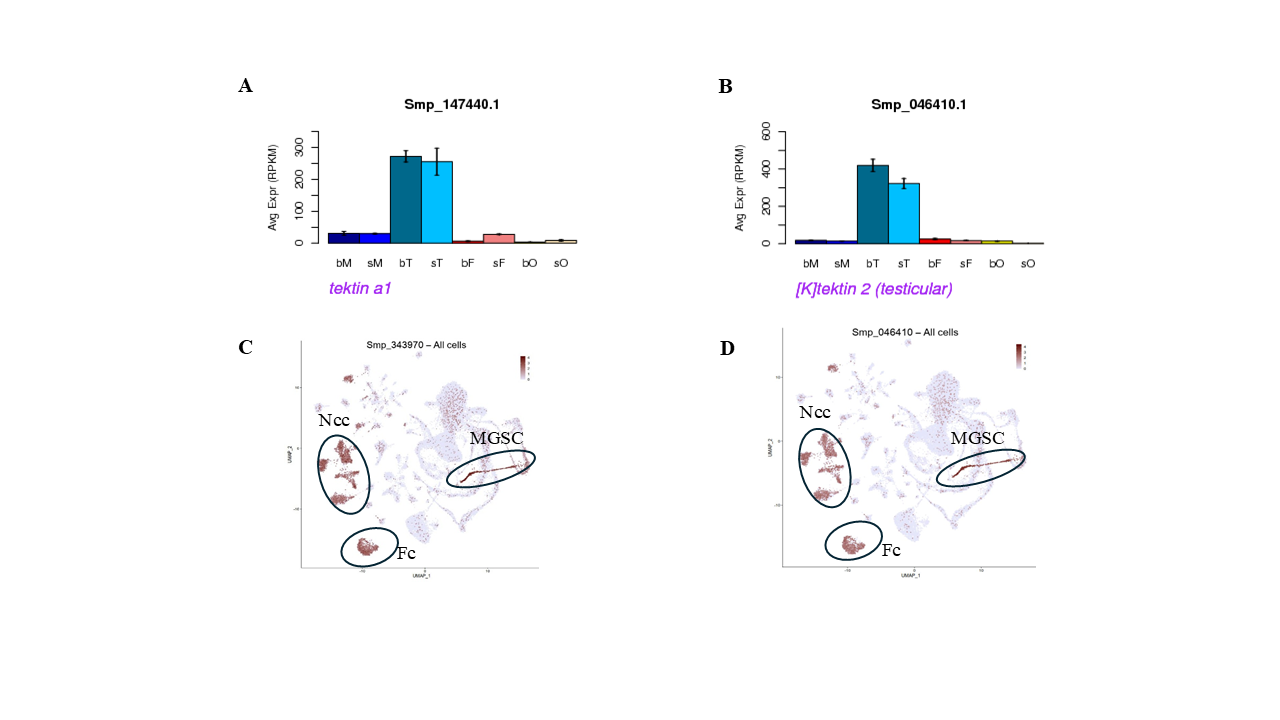

Supplement: S14 Fig — Previous bulk RNA-seq data of adult S. mansoni and their gonads (42) showed testis-preferential expression in tektin a1 (A; previous gene number: Smp_147440; according to the new annotation: Smp_343970) and tektin 2 (B; Smp_046410). C-D, Single cell RNA-seq data exhibited dominant expression for both tektins in neuronal cell clusters 2-6 and 30 (Ncc), flame cells Fc, and late male germ cells (MGSC) (56). Abbreviations: bM, males with pairing experience; sM, males without pairing experience; bT, testes of bM; sT, testes of sM; bF, females with pairing experience; sF, females without pairing experience; bO, ovaries of bF; sO, ovaries of sF; NCC, neuronal cell cluster; Fc, flame cell. (TIF) [file ppat.1014096.s014.tif]
